# Energy Constrained Optimization for Spreading Factor Allocation in LoRaWAN

**DOI:** 10.3390/s20164417

**Published:** 2020-08-07

**Authors:** Shusuke Narieda, Takeo Fujii, Kenta Umebayashi

**Affiliations:** 1 Graduate School of Engineering, Mie University, Mie 514–8507, Japan; 2 Advanced Wireless Communication Research Center, The University of Electro–Communications, Tokyo 182–8585, Japan; fujii@awcc.uec.ac.jp; 3 Graduate School of Engineering, Tokyo University of Agriculture and Technology, Tokyo 183–8538, Japan; ume_k@cc.tuat.ac.jp

**Keywords:** LoRaWAN, spreading factor, energy consumption

## Abstract

This paper discusses a spreading factor allocation for Long Range Wide Area Network (LoRaWAN). Because Long Range (LoRa) is based on chirp spread spectrum that each spreading factor is approximately orthogonal to each other, the performance of LoRaWAN can be enhanced by allocating the spreading factor appropriately to end devices (EDs). Several spreading factor allocation techniques have been reported. Techniques shown in existing studies can improve some characteristics (e.g. throughput or packet reception probability (PRP)); however, there are a few studies that have focused on the energy consumption of the EDs. The LoRa communication offers a low power communication and this enables the improvement of the performance in exchange for the energy consumption. This paper presents a performance improvement technique via spreading factor allocations for LoRaWAN. We define the optimization problem for the spreading factor allocation to maximize the PRP under a constraint for the average energy consumption of all the EDs. It enables for the performance improvement under the constraint of the average energy consumption of all the EDs by solving the problem. This study further develops a method to solve the defined problem based on a distributed genetic algorithm, which is metaheuristics method. Although the techniques shown in the existing studies give the average energy consumption as a result of the performance improvement by the spreading factor allocation, the presented technique can enhance the LoRaWAN performance by allocating the spreading factor to EDs under the constraint for the average energy consumption of all the EDs. Numerical examples validate the effectiveness of the presented technique. The PRP performance of the presented technique is superior to that of the techniques shown in the existing studies despite that the average energy consumption of all the EDs of the presented technique is less than that of the techniques shown in the existing studies.

## 1. Introduction

Recently, applications for the Internet of Things (IoT) have been considerably focused and a lot of IoT devices will be deployed to gather information for smart metering, environmental monitoring, and health-care applications. It is well known that low-power wide-area (LPWA) networks (LPWANs) are communication systems that are supported by massive IoT devices with a low traffic end device (ED) [1]. Further, LPWAN technologies can provide a low cost for EDs and the production of infrastructures. LPWA communication systems enable a long range communication. In addition, LPWANs can provide the communication infrastructure that builds wireless sensor networks (WSNs) due to their characteristics. Some LPWA technologies, such as SigFox [2], NB-IoT [3], and Long Range Wide Area Network (LoRaWAN) [4], operate in the sub-GHz band. Among these technologies, it is presumed that such IoT systems can be developed based on LoRaWAN because LoRaWAN can be used to build a private/local network in an unlicensed band. Further, several studies and evaluations for LoRaWAN have been reported; for WSNs [5], a smart city scenario [6,7], smart golf course [7], evaluations in university campus [8], design and implementation for LoRaWAN system with edge computing [9], human centric health and wellness monitoring applications [10], and so on.

Long Range (LoRa) communications employ chirp spread spectrum (CSS) modulation [11], which is one of spread spectrum, as a modulation scheme. Traditionally, the LoRa modulations have been analyzed from the viewpoint of signal processing [12] and have been derived a closed-form for a bit error rate (BER) representation in additive white Gaussian noise (AWGN) channel, Rayleigh fading channels [13] and Nakagami fading channel [14]. Six types of spreading factor, which are capable of spreading code, are generally used and allocated to LoRa EDs. The difference in the spreading factor causes the difference of the time on air (ToA) and sensitivity. Although the spreading factors in LoRa are not perfectly orthogonal [15,16,17], LoRa communications can provide a rough interference avoidance capability, which enables multiple concurrent transmissions of some asynchronized signals modulated with different spreading factors.

The LoRa communications offer a low power communication and this enables the performance improvement in exchange for the energy consumption. Therefore, the management of energy consumption is very important issue for LoRaWAN. The spreading factor strongly affects the energy consumption of EDs because the ToA for each spreading factor is different. It can be considered that the performance of LoRaWAN can be enhanced to allocate the spreading factor appropriately to EDs. However, the energy consumption has never been much considered in the spreading factor allocation technique.

This paper presents a spreading factor allocation technique that considers the energy consumption at EDs for the performance improvement of LoRaWAN. In order to allocate the spreading factor appropriately, a constrained optimization problem is defined. For this problem, the minimum packet reception probability (PRP) of all the EDs is maximized under a constraint for the average energy consumption of all the EDs. This study attempts to allocate an appropriate spreading factor to EDs by solving the problem. The constrained optimization problem defined in this paper can be regarded as the combination optimization problem, and it is difficult to solve the problem by convex optimization [18]. To overcome this, this study attempts to use distributed genetic algorithm (GA) [19,20,21]. Distributed GA is one of the metaheuristics methods and diversity preservation methods based on spatial separation to avoid premature convergence. In distributed GA, the population is divided into several subpopulations, and each one of them being processed by GA, independently of the others. Distributed GA attempts to improve premature convergence by preserving diversity due to the semi isolation of the subpopulations. We develop a solving method based on distributed GA for the defined constrained optimization problem.

The remainder of this paper is organized as follows. Section 2 shows the related works of the presented technique. Section 3 shows the fundamental system model of LoRaWAN. In Section 4, we formulate the constrained optimization problem for the performance improvement of EDs, and the solving method based on distributed GA is shown in Section 5. Numerical examples are shown in Section 6. Finally, we conclude the paper in Section 7.

## 2. Related Works

Traditionally, spreading factor allocation techniques for the EDs in LoRaWAN have been reported [17,22,23,24,25,26,27,28]. In [17], a simple spreading factor allocation technique based on the distance between each ED and the LoRa gateway (GW) has been introduced. Aimed at throughput maximization, the spreading factor that is allocated to the ED is determined based on the distance between each ED and GW, the width of the LoRaWAN, and the path loss exponent [22]. This is based on an analysis for the imperfect spreading factor orthogonality [17]. Furthermore, the spreading factor allocation algorithm based on the matching theory to maximize the minimum throughput of the ED has been presented [22]. For the purpose of the minimizing of the collision probability, the spreading factor allocation with transmission power control has been presented [23]. In [24], the spreading factor allocation for determining the geographical area for each spreading factor has been presented. There is a fair and an unfair allocation algorithm of the spreading factor to equally divide the spreading factor between the EDs while respecting the received signal strength intensity (RSSI) values and ToA [25]. In [26], a tree-based spreading factor clustering algorithm for LoRaWAN ensures that the connectivity has been presented. K-means clustering based allocation algorithm for a large scale LoRaWAN has been presented [27]. For simultaneous transmissions of EDs with the same spreading factor, the LoRa GW can still decode the stronger transmission. This is known as capture effect. Under considering the imperfect orthogonality and capture effect, optimal spreading factor allocation has been considered [28]. Thus, there are several targets for optimization with the allocation techniques shown in the existing studies. Among them, the maximizing the minimum packet reception probability (PRP) is suitable for the WSN in environmental monitoring. This is because it is important to collect observations from as many EDs as possible. These are summarized in Table 1.

In the existing studies [17,22,23,24,25,26,27,28], the performance can be enhanced in exchange for the energy consumption. In [29,30], spreading factor allocation techniques considering on energy have been presented. However, the references [29,30] only consider the energy efficiency and the optimization of the LoRaWAN performance is not considered. The presented technique is effective for the design of LoRaWAN that can demonstrate the best performance under the constraint for the average energy consumption of all EDs. Further, although the transmission power control affects the energy efficiency at each ED, the effect of the transmission power control is less than that of the spreading factor allocation. For example, the current consumption changes about 1.5 times when the transmission power is changed from −2 dBm to 10 dBm, while the current consumption changes about 32 times when the spreading factor is changed from 7 to 12. This can be obtained by Semtech LoRa Calculator [31]. Therefore, we focus on the spreading factor allocation in this paper, and the optimization for the spreading factor allocation including the transmission power control is a future work.

The contribution of this paper is to provide the design method of LoRaWAN enabling the improvement of LoRaWAN performances by allocating the spreading factor to EDs under the limited average energy consumption of all the EDs.

## 3. Preliminary Notion

### 3.1. LoRa and LoRaWAN

This paper considers the LoRaWAN which is composed of $N_{\mathrm{ED}}$ EDs and one LoRa GW. The LoRa GW is located in the center of the communication areas of LoRaWAN with a radius *R* m. The ED has some physical sensors and it transmits the observed data. The LoRa GW collects the transmitted data from the EDs. In this paper, we do not assume acknowledgment from the LoRa GW to each ED, i.e., the EDs only transmit their data to the LoRa GW. The LoRa modulation is based on the CSS which is one of the spread spectrum communications, and we assume that the LoRa modulation uses the six types of spreading factors $s_{i}$ ($s_{i}\in S=\left\{7,\cdots ,12\right\},i=1,\cdots ,N_{\mathrm{ED}}$) [32]. The difference in the spreading factor causes the difference of the sensitivity and the ToA. Table 2 shows an example of the sensitivity and ToA for all spreading factors in the LoRa communication with a 125 kHz bandwidth, coding rate 4/5 (parity check), 20 byte payload data, and 8 symbols from the packet preambles. Note that these values are obtained by Semtech LoRa Calculator [31]. Further, the CSS characterizes LoRa modulation signals that have some interference avoidance capabilities. However, this is not perfect because of imperfect orthogonality of the spreading factor [17]. To represent the imperfectness of the orthogonality of the spreading factor, some examples have been reported [16,33]. The imperfectness can be represented as a square matrix. Let M denote a |S| dimension square matrix that represents a signal-to-interference ratio (SIR) matrix. Note that the unit of each element in the matrix is decibel. Among the traditional SIR matrices, this study employed M which is obtained via an actual measurement, and it is given by [16].

(1) $$\begin{array}{c} M=\left[\begin{array}{cccccc} 1 & -8 & -9 & -9 & -9 & -9\\ -11 & 1 & -11 & -12 & -13 & -13\\ -15 & -13 & 1 & -13 & -14 & -15\\ -19 & -18 & -17 & 1 & -17 & -18\\ -22 & -22 & -21 & -20 & 1 & -20\\ -25 & -25 & -25 & -24 & -23 & 1 \end{array}\right]. \end{array}$$

Note that whether or not almost concurrently transmitted packets collide is decided based on the matrix. Concretely, in order to judge the collision of a packet, the comparison with all other packets existing in the air at the same time is executed based on the matrix. Further, the matrix includes the capture effect.

### 3.2. Channel Model

The received signal power of the LoRa GW from the *i*th ED $P_{\mathrm{RX},i}$ can be written as

(2) $$\begin{array}{c} P_{\mathrm{RX},i}=P_{\mathrm{TX},i}-L_{i} \end{array}$$

where $P_{\mathrm{TX},i}$ is a transmission power at *i*th ED and $L_{i}$ is the path loss between the LoRa GW and *i*th ED and $L_{i}=-10\log_{10}\left(d_{i}^{\alpha }f_{c}^{2}\times 10^{-2.8}\right)$ [17] where α and $f_{c}$ are the path loss exponent and the carrier frequency, respectively.

## 4. Problem Formulation

In this section, the optimization problem is formulated, which maximizes the minimum PRP of the ED under the constraint for the average energy consumption of all the EDs. In this paper, we assume that all EDs are a static node and the assumption holds in many cases for WSNs. Furthermore, we assume that the number of EDs in the network does not change. In this section, first, the PRP is derived while considering the imperfect orthogonality of the spreading factor in LoRaWAN. Next, the simple energy consumption model of the ED is discussed. From these, a constrained optimization problem was formulated to maximize the minimum PRP under the constraint for the energy consumption. Finally, the distributed GA based algorithm is discussed to solve the problem.

### 4.1. Packet Reception Probability

In this subsection, we derive the PRP while considering the imperfect orthogonality of the spreading factor in LoRaWAN. If all spreading factors are perfectly orthogonal, no packet collision occurs between the EDs with the different spreading factors. Actually, all of the spreading factors are imperfectly orthogonal and the packet collision occurs, even for the EDs with different spreading factors. Therefore, the imperfectness determines the number of EDs that affects the packet collision. Let s denote the $N_{\mathrm{ED}}$ dimensional vector for the allocated spreading factor to the $N_{\mathrm{ED}}$ EDs. Although the duty cycle is determined in LoRaWAN, we assume that the packet generation roughly follows Poisson distribution. Then, the PRP at *i*th ED $P_{\mathrm{PRP},i}\left(s\right)$ can be written as

(3) $$\begin{array}{c} P_{\mathrm{PRP},i}\left(s\right)={\left(1-\frac{1}{T_{i}}\right)}^{2T_{s_{i}}\left\{N_{i}\left(s\right)-1\right\}},\;i=1,\cdots ,N_{\mathrm{ED}}, \end{array}$$

where $T_{i}$, $T_{s_{i}}$, $N_{i}\left(s\right)$ are an average packet transmission interval at *i*th ED, the ToA of the spreading factor $s_{i}$ and the number of the EDs that can cause the packet collision at *i*th ED. $N_{i}\left(s\right)$ is given by

(4) $$\begin{array}{c} N_{i}\left(s\right)=1+\sum_{k=1,k\neq i}^{N_{\mathrm{SUC}}\left(s\right)}H_{1}\left(m_{s_{i},s_{k}},L_{i},L_{k}\right),\;i=1,\cdots ,N_{\mathrm{ED}}, \end{array}$$

where $N_{\mathrm{SUC}}\left(s\right)$, $m_{s_{i},s_{k}}$ and $H_{1}\left(\cdot ,\cdot ,\cdot \right)$ are the number of EDs that satisfy the sensitivity, the $s_{i}$ columns and the $s_{k}$ rows component of the SIR matrix M defined in Equation (1), and the function defined as follows,

(5) $$\begin{array}{c} H_{1}\left(m_{s_{i},s_{k}},L_{i},L_{k}\right)=\left\{\begin{array}{cc} H\left[m_{s_{i},s_{k}}<\left(L_{i}-L_{k}\right)\right] & L_{i}\geq P_{s_{i}}\\ 0 & L_{i}<P_{s_{i}} \end{array}\right.,\;i=1,\cdots ,N_{\mathrm{ED}},\;k=1,\cdots ,N_{\mathrm{ED}} \end{array}$$

where $H\left[X\right]$ is the function which returns 1 for X is true and 0 for X is false. Further, $N_{\mathrm{SUC}}\left(s\right)$ can be written as

(6) $$\begin{array}{c} N_{\mathrm{SUC}}\left(s\right)=\sum_{i=1}^{N_{\mathrm{ED}}}H_{2}\left(P_{s_{i}},P_{\mathrm{TX},i},L_{i}\right) \end{array}$$

where $P_{s_{i}}$ and $H_{2}\left(\cdot ,\cdot ,\cdot \right)$ are the sensitivity for the spreading factor $s_{i}$ and the function defined as follows,

(7) $$\begin{array}{c} H_{2}\left(P_{s_{i}},P_{\mathrm{TX},i},L_{i}\right)=\left\{\begin{array}{cc} H\left[P_{s_{i}}<\left(P_{\mathrm{TX},i}-L_{i}\right)\right] & L_{i}\geq P_{s_{i}}\\ 0 & L_{i}<P_{s_{i}} \end{array}\right.,\;i=1,\cdots ,N_{\mathrm{ED}}. \end{array}$$

As shown in these equations, $N_{\mathrm{SUC}}\left(s\right)$, i.e., $P_{\mathrm{PRP},i}\left(s\right)$ strongly depends on the allocated spreading factors and the SIR matrix shown in Equation (1). The appropriately allocated spreading factor can improve $P_{\mathrm{PRP},i}\left(s\right)$ whereas the inappropriately allocated spreading factor degrades $P_{\mathrm{PRP},i}\left(s\right)$. Therefore, the appropriate allocation of the spreading factors in LoRaWAN allows multiple access with the concurrent transmissions even if they are imperfectly orthogonal.

### 4.2. Simple Energy Consumption Model

In this subsection, we describe the energy consumption model of the EDs that are employed in this paper. As shown in the previous section, we assume the simple LoRaWAN model where all of the EDs only transmit observed data without acknowledgement. In order to focus the optimization for the spreading factor allocation in this paper, we assume a simple energy consumption model, which is composed of a data transmission and a sleep mode. Let $I_{\mathrm{TX}}$ and $I_{\mathrm{SLP}}$ denote the current for the data transmission and sleep mode, respectively. Then, the average current consumption $W\left(s\right)$ mAh for all of the LoRa EDs for an hour can be written as

(8) $$\begin{array}{cc} W\left(s\right) & =\frac{3600}{N_{\mathrm{ED}}}\sum_{i=1}^{N_{\mathrm{ED}}}\frac{T_{s_{i}}}{T_{i}}I_{\mathrm{TX}}+\left(1-\frac{T_{s_{i}}}{T_{i}}\right)I_{\mathrm{SLP}}. \end{array}$$

Note that $I_{\mathrm{TX}}$ and $I_{\mathrm{SLP}}$ used the calculated values by Semtech LoRa Calculator [31]. Table 3 shows the current consumption values for the 10 dBm transmission power obtained by Semtech LoRa Calculator. These values are the current consumption of SX1272, which is a LoRa transceiver produced by Semtech. We employ the simple energy consumption model based on Table 3 in conjunction with Table 2 as shown in the previous section.

### 4.3. Optimization Problem

In order to take into account the energy consumption of EDs in the spreading factor allocation, we formulate the optimization problem which is the maximization of the minimum PRP of the ED under the constraint for the average energy consumption of all the EDs. In this paper, we define the following constrained optimization problem using $P_{\mathrm{PRP},i}\left(s\right)$ defined in Equation (3) as follows,

(9) $$\begin{array}{c} \begin{array}{cc} s^{\mathrm{OPT}}= & \underset{s\in S}{\arg\;\max}\min_{i}\left\{P_{\mathrm{PRP},i}\left(s\right)\right\},\;i=1,\cdots ,N_{\mathrm{ED}}\\ \; & \mathrm{subject}\;\mathrm{to}\;W\left(s\right)\leq W_{C},\;P_{s_{i}}\leq P_{\mathrm{RX},i},\;\forall i, \end{array} \end{array}$$

where $s^{\mathrm{OPT}}$ and $W_{C}$ are the $N_{\mathrm{ED}}$ dimensional vector for the optimal allocated spreading factor ($s^{\mathrm{OPT}}=\left[s_{1}^{\mathrm{opt}},\cdots ,s_{N_{\mathrm{ED}}}^{\mathrm{opt}}\right]$ where $s_{i}^{\mathrm{opt}}$ is an optimum spreading factor for *i*th ED) and a constraint for the average current consumption of all the EDs, respectively. Note that $P_{s_{i}}\leq P_{\mathrm{RX},i},\forall i$ in Equation (9) represents a constraint for the sensitivity, i.e., whether the allocated spreading factor for the EDs satisfies the sensitivity or not. The problem is a combinational optimization problem, and the solution cannot be obtained with the convex optimization technique [18]. We show how to solve this problem in the next section.

## 5. Distributed Genetic Algorithm Based Solving Method

In this section, algorithms for the optimization problem that are defined in Equation (9) are discussed. Metaheuristics method [34], which includes tabu search, genetic programming or GA, is frequently employed to solve the combinational optimization problem. It is well known that the metaheuristics method can provide a sufficiently good solution to the problem. GA [35] is one of the evolutionary algorithms that are included in artificial intelligence technology. GA is a global search technique where it does not use the gradient of the cost function. However, the limitation of GA is that it lacks a fine local tuning capability and it also requires a large computation time. Therefore, this study attempts to solve the combinational optimization problem as shown in Equation (9) by distributed GA [19]. In distributed GA, the population is divided into $N_{\mathrm{IS}}$ the subpopulation which is called island where $N_{\mathrm{IS}}$ is the number of island, and GA is executed in each island to search the optimal solution. The search results for each island are shared among all of the islands by exchanging selected individuals between each islands at some generations (migration intervals), which is known as migration. Distributed GA is known as a search method that has the characteristics of a local search method while being a global search method because an optimal solution is searched for each island.

**Figure 1:**
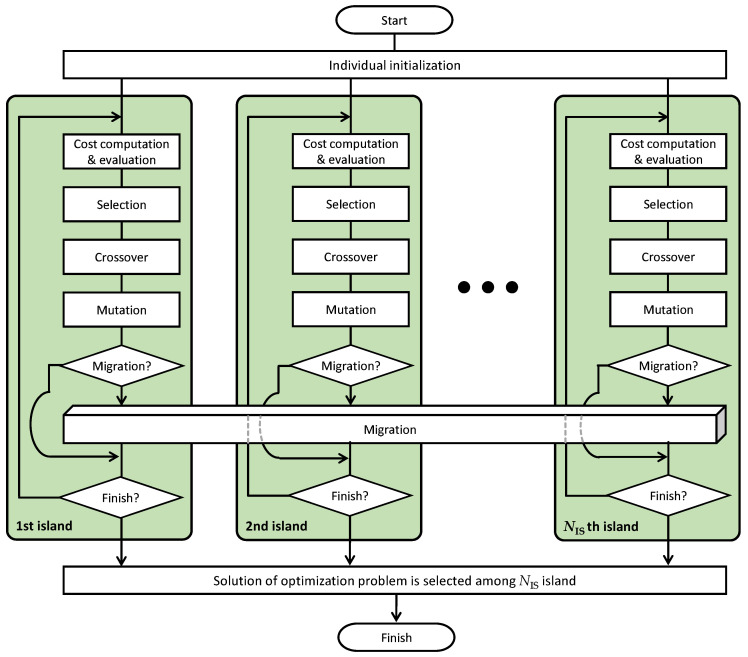
Overview of distributed genetic algorithm (GA).

The overview of distributed GA is shown in Figure 1. This study employed two point crossover scheme and elite selection scheme as crossover and selection in Figure 1, respectively. In the individual initialization shown in Figure 1, each individual for the representation of the spreading factor is initialized. As an individual, the $N_{\mathrm{ED}}$ dimensional vector is used, which is the vector of the real-coded allocated spreading factor. Let $s_{l,j}$ denote the $N_{\mathrm{ED}}$ dimensional vector for the allocated spreading factors, which represents the *j*th individual in the *l*th island. Each individual is randomly initialized by the spreading factor satisfying the sensitivity of each ED. Let $I_{\mathrm{MIN}}$ denote an average current consumption given by the minimum spreading factor that satisfies the sensitivity. To improve the convergence characteristics, individuals that the minimum value of the spreading factors satisfying the sensitivity, i.e., the average energy consumption of the individual is $I_{\mathrm{MIN}}$, are included in the population. In cost computation in Figure 1, the cost value $C_{l,j}$ included in the *j*th individual of the *l*th island is calculated. The defined problem shown in Equation (9) is not a multiobjective optimization but the constrained optimization problem. Because the result does not satisfy the constraints, it cannot be employed as a solution for Equation (9). As a result, it is difficult to employ GA or distributed GA as it is as the solution to the problem. In this paper, we attempt to employ the indicator function for the description of the constraint. We let I(X) denote the indicator function (I(X)=1 when X≥0 and I(X)=0 when X<0). Based on these, the cost value $C_{l,j}$ can be written as

(10) $$\begin{array}{cc} C_{l,j} & =1-\min_{i}\left\{P_{\mathrm{PRP},i}\left(s_{l,j}\right)\right\}+I\left[W\left(s_{l,j}\right)-W_{C}\right]+\sum_{i=1}^{N_{\mathrm{ED}}}I\left[P_{s_{i}^{\left(l,j\right)}}-P_{\mathrm{RX},i}\right],\\ \; & \;l=1,\cdots ,N_{\mathrm{IS}},\;j=1,\cdots ,\left\lfloor N_{I}/N_{IS}\right\rfloor , \end{array}$$

where $N_{I}$, $s_{l,j}$ and $s_{i}^{\left(l,j\right)}$ are the number of individuals among all of the island, $N_{\mathrm{ED}}$ dimensional spreading factor vector for *j*th individual in the *l*th island and the *i*th gene for the $s_{l,j}$, respectively. The sum of the first term and the second term in Equation (10) never exceeds one because the second term represents the probability. The cost $C_{l,j}$ can be taken $0\leq C_{l,j}\leq 1$ for $W\left(s_{l,j}\right)\leq W_{C}$ and all of the EDs allocated by some spreading factors that satisfy the sensitivity. Because elite selection scheme is employed, the selected individual is given by

(11) $$\begin{array}{c} l_{\mathrm{EL}},j_{\mathrm{EL}}=\underset{l,j}{\arg\;\min}C_{l,j},\;l=1,\cdots ,N_{\mathrm{IS}},\;j=1,\cdots ,\left\lfloor N_{I}/N_{\mathrm{IS}}\right\rfloor , \end{array}$$

where $l_{\mathrm{EL}}$ and $j_{\mathrm{EL}}$ are the island number and the individual number for the individual having the lowest cost value, respectively. Finally, the solution of the constrained optimization problem can be written as

(12) $$\begin{array}{c} {\hat{s}}^{\mathrm{OPT}}=\left\{\begin{array}{cc} s_{l_{\mathrm{EL}},j_{\mathrm{EL}}} & 0\leq C_{l_{\mathrm{EL}},j_{\mathrm{EL}}}\leq 1\\ \mathrm{none} & \mathrm{others} \end{array}\right., \end{array}$$

where ${\hat{s}}^{\mathrm{OPT}}$ is $N_{\mathrm{ED}}$ dimensional suboptimal allocated spreading factor vector. Note that “none” in Equation (12) means that any individuals are not satisfied to the constraint. The decision is executed after the specified number of generations, and the algorithm determines whether there is no solution in this case.

## 6. Numerical Example

### 6.1. Parameter Setup

In this section, we show some numerical examples for the evaluation of the presented technique. All numerical examples shown in this section were obtained by computer simulations using MATLAB 2019b. Table 4 lists the parameters that were employed for the computer simulation. These parameters were driven from EU863-870 ISM Band from document [36]. The communication area of LoRaWAN was within the circle with R=6000 m, 10,000 m, and the $N_{\mathrm{ED}}$ EDs were uniformly distributed. The path loss exponent between the EDs and the GW was 2.7 (suburban) [24]. Each ED transmitted its own observation with the $P_{\mathrm{TX},i}=10$ dBm, ∀i transmission power, a 125 kHz bandwidth, $T_{i}=360\;s/1\;\mathrm{packet},\forall i$ transmission interval and the duty cycle <1%. ToA and sensitivity employed in the simulation are listed in Table 2. As shown in previous section, parameters in Table 2 were derived from Semtech LoRa Calculator [31] under 125 kHz bandwidth, coding rate 4/5 (parity check), 20 byte payload data and 8 symbols from the packet preabmles. For the parameters of distributed GA, the number of individuals and islands were set to 128 and 16, respectively. The results presented in this section were obtained by iterating the developed algorithm for 2000 generations. After 2000 generations, the decision for the final solution shown in Equation (12) was executed. Further, a packet error rate (PER), which could be derived from the BER of LoRa communications, was considered for the simulation. The BER $P_{\mathrm{BER},i}$ in AWGN channel for *i*th ED is given by [13]

(13) $$\begin{array}{c} P_{\mathrm{BER},i}\approx 0.5Q\left(\frac{\sqrt{\Gamma_{i}\cdot 2^{s_{i}}}-{\left\{{\left(H_{2^{s_{i}}-1}\right)}^{2}-\frac{\pi^{2}}{12}\right\}}^{1/4}}{\sqrt{H_{2^{s_{i}}-1}-{\left\{{\left(H_{2^{s_{i}}-1}\right)}^{2}-\frac{\pi^{2}}{12}\right\}}^{1/2}+0.5}}\right) \end{array}$$

where $\Gamma_{i}$, $H_{m}\approx \ln\left(m\right)+1/\left(2m\right)+0.57722$ and $Q\left(z\right)=\frac{1}{\sqrt{2\pi }}\int_{z}^{\infty }\exp\left[-\frac{u^{2}}{2}\right]du$ are the SNR, the harmonic number and the Q-function, i.e., the tail function of the standard normal distribution, respectively. In this section, the presented technique, the minimum energy which is given by the minimum spreading factor satisfying the sensitivity of each ED and two techniques, i.e., (i) the allocation technique considering on the interference between each ED [24] (SF-SIR) and distance based allocation [17] (SF-distance), are compared.

### 6.2. Results; Changing of the Number of ED ($N_{\mathrm{ED}}$), R=6000 m

**Figure 2:**
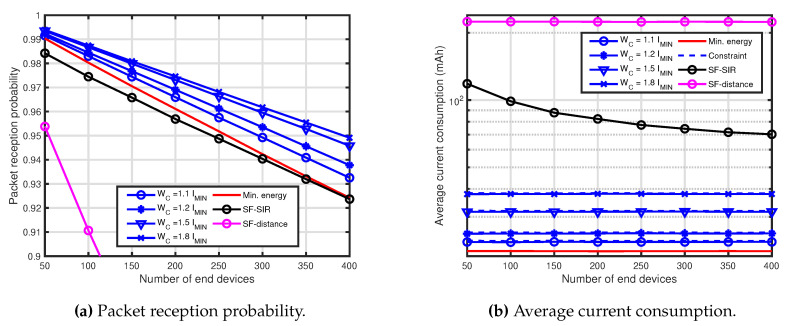
Performance comparison for R=6000 m, $W_{C}=1.1I_{\mathrm{MIN}}$ mAh, $1.2I_{\mathrm{MIN}}$ mAh, $1.5I_{\mathrm{MIN}}$ mAh and $1.8I_{\mathrm{MIN}}$ mAh.

Figure 2 shows the performance comparison results for R=6000 m. In Figure 2, $W_{C}=1.1I_{\mathrm{MIN}}$ mAh, $1.2I_{\mathrm{MIN}}$ mAh, $1.5I_{\mathrm{MIN}}$ mAh and $1.8I_{\mathrm{MIN}}$ mAh were employed for the presented technique. Figure 2a shows the PRP of the presented technique, the SF-SIR and the SF-distance. The performance of the presented technique outperforms that of the SF-SIR and the SF-distance, and the performance increased as the energy consumption increased. However, the performance for the $W_{C}=1.5I_{\mathrm{MIN}}$ mAh was not as much improved as the constraint. The reason for this is shown in later. Comparing with the minimum energy and the SF-SIR, the performance of $I_{\mathrm{MIN}}$ (min. energy) was superior to that of the SF-SIR and the SF-distance. Further, comparing with two techniques, the performance of the SF-SIR outperformed that of the SF-distance. Figure 2b shows the average current consumption of all of the EDs of the presented technique and the other techniques. The current consumptions of the presented technique maintained each constraint, and the current consumption of the presented technique was lower than that of the other techniques. From these, it can be seen that the presented technique could improve the performance under the constraint for the average current consumption of all the EDs.

**Figure 3:**
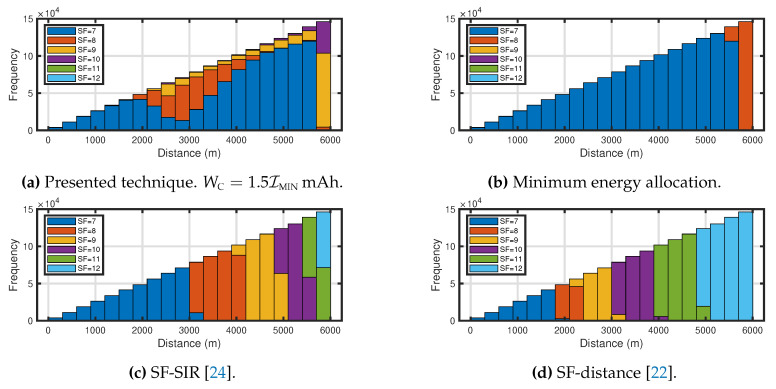
Histogram of allocated spreading factor for R=6000 m, $N_{\mathrm{ED}}=150$.

Figure 3 shows histograms of the allocated spreading factors for the distance between the ED and GW, are shown. In Figure 3, the performance of the presented technique with $W_{C}=1.5I_{\mathrm{MIN}}$ mAh, the minimum energy allocation, the SF-SIR and the SF-distance are shown for $N_{\mathrm{ED}}=150$. Figure 3a shows a histogram of allocated spreading factor by the presented technique. The spreading factor 7 was allocated to a lot of EDs for overall of the communication areas. Based on the discussion in the previous section, it can be considered that the characteristics for the distance distribution of the allocated spreading factor depended on the SIR matrix that was defined in Equation (1) and the sensitivity of the LoRa communications. Figure 3b–d show histograms of allocated spreading factor by the minimum energy allocation, the SF-SIR and the SF-distance, respectively. It can be seen that the SF-SIR and the SF-distance allocated more large spreading factor than the presented technique and the minimum energy allocation.

Table 5 and Table 6 show the PRP and average current consumption according to the distance between the LoRa GW and each ED for $N_{\mathrm{ED}}=150$, respectively. As shown in Table 5, it can be seen that the farther the distance between the LoRa GW and the ED, the more the performance of the PRP deteriorated. The similar tendency can be seen in all $W_{C}$ values. It can be seen that the tendency depends on the allocated spreading factor to each ED as shown in Figure 3a. As shown in Table 6, it can be seen that the farther the distance, the higher the average current consumption. The tendency in Table 6 is similar to that in Table 5. Furthermore, it can be seen that the average current consumptions for each $W_{C}$ maintain each constraint.

To evaluate convergence characteristics of the presented technique, we discuss a computation time of the presented spreading factor allocation technique. Table 7 shows the computation time to obtain the allocated spreading factor for $N_{\mathrm{ED}}$ EDs averaged by 100 trials. Results were obtained by the computer with specifications: Intel(R) Xeon(R) Gold 6140 CPU@2.30 GHz, memory size was 64 GB. Note that parallel computations by any MATLAB toolbox were not employed to obtain the results shown in Table 7 for the evaluation of the presented technique. As shown in Table 7, it can be seen that the computation time of the presented technique did not take much time. In addition, because the presented algorithm is based on distributed GA suitable for the computation on a graphics processing unit (GPU), it can be said that further speeding up of the implemented presented algorithm can be expected.

### 6.3. Results; Changing of the Number of ED ($N_{\mathrm{ED}}$), R=10,000 m

**Figure 4:**
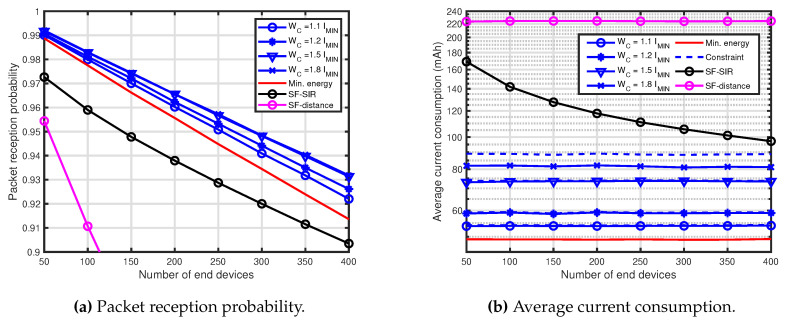
Performance comparison for R=10,000 m, $W_{C}=1.1I_{\mathrm{MIN}}$ mAh, $1.2I_{\mathrm{MIN}}$ mAh, $1.5I_{\mathrm{MIN}}$ mAh and $1.8I_{\mathrm{MIN}}$ mAh.

Figure 4 shows the performance comparison results for R=10,000 m. In Figure 4, $W_{C}=1.1I_{\mathrm{MIN}}$ mAh, $1.2I_{\mathrm{MIN}}$ mAh, $1.5I_{\mathrm{MIN}}$ mAh and $1.8I_{\mathrm{MIN}}$ mAh were employed for the presented technique. Figure 4a shows the PRP of the presented technique, the SF-SIR and the SF-distance. Although the tendency of the performances was almost the same as Figure 2a, the performance of the high $W_{C}$ for R=10,000 m was not much more improved than that for R=6000 m. It can be seen that the effect of the performance improvement due to the increase in average current consumption decreased as the communication area increased.

### 6.4. Results; Changing of the Radius of Communication Areas (*R*), $N_{\mathrm{ED}}=150$

**Figure 5:**
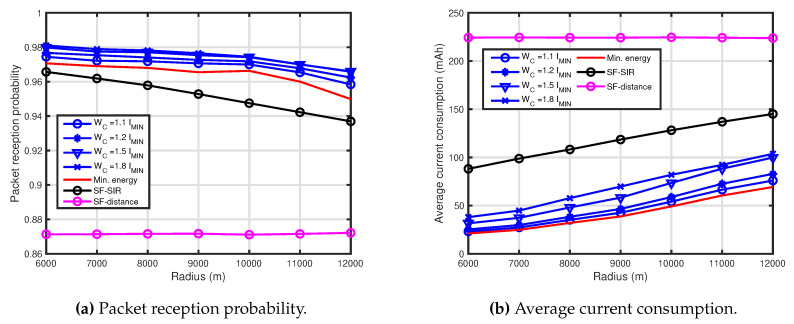
Performance of the presented technique for some radii of the communication area. $N_{\mathrm{ED}}=150$, $W_{C}=1.1I_{\mathrm{MIN}}$ mAh, $1.2I_{\mathrm{MIN}}$ mAh, $1.5I_{\mathrm{MIN}}$ mAh and $1.8I_{\mathrm{MIN}}$ mAh.

Figure 5 shows characteristics in some radii of the communication area. In Figure 5, the presented technique and the other techniques are compared for $N_{\mathrm{ED}}=150$, $W_{C}=1.1I_{\mathrm{MIN}}$ mAh, $1.2I_{\mathrm{MIN}}$ mAh, $1.5I_{\mathrm{MIN}}$ mAh and $1.8I_{\mathrm{MIN}}$ mAh. Figure 5a,b show the PRP and the average current consumption of the presented technique, the SF-SIR and the SF-distance, respectively. As shown in Figure 5, it can be also seen that the presented technique is effective.

### 6.5. Results; Changing of the Number of EDs ($N_{\mathrm{ED}}$), R=6000 m, with Changing of Quality of Communication Link between LoRa GW and Each ED

**Figure 6:**
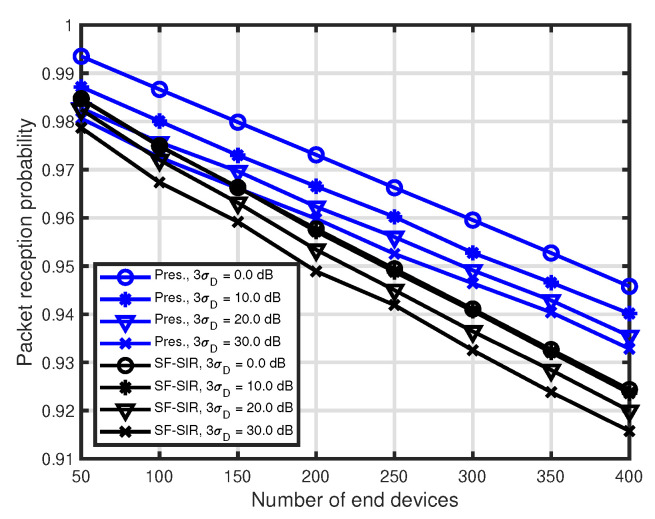
Performance comparison when the quality of the communication link is changed. R=6000 m, $W_{C}=1.5I_{\mathrm{MIN}}$ mAh, $3\sigma_{D}=0$ dB, 5 dB, 10 dB, 15 dB.

Finally, we evaluate the presented allocation technique for the environment that the quality of the communication link between the LoRa GW and some EDs is changed. Although all EDs are assumed as a static node, the quality of the communication links between the LoRa GW and some EDs may have changed due to the environmental changes around the EDs. To represent this, we employed Gaussian random variable with zero mean and standard deviation $\sigma_{D}$, i.e., the random variable is added to the original RSSI value. We let ${\tilde{L}}_{i}$ denote the path loss after changing the quality of the communication link and ${\tilde{L}}_{i}=L_{i}+N\left(0,\sigma_{D}^{2}\right)$. $L_{i}$ in Equation (2) is substituted to ${\tilde{L}}_{i}$ in this subsection. Figure 6 shows the performance of the PRP of the presented technique for R=6000 m, $W_{C}=1.5I_{\mathrm{MIN}}$ mAh and $3\sigma_{D}=0$ dB, 5 dB, 10 dB, 15 dB. The quality of the link between the LoRa GW and randomly selected 10% of the EDs is changed in the simulation period. Figure 6 shows the performance of the PRP. It can be seen that the performance of the presented technique does not deteriorate so much for the deviation in comparison with that of the SF-SIR.

## 7. Conclusions

This paper presented the spreading factor allocation technique in LoRaWAN. To allocate the spreading factor appropriately, the energy consumption constrained optimization problem was defined. The defined problem is a combinational optimization problem, and it is non-convex optimization problem. We developed a distributed GA based algorithm for the solution of the problem. The presented technique can enhance the LoRaWAN performance by allocating the spreading factor to EDs under the constraint for the average energy (current) consumption of all the EDs. Numerical examples were shown to validate the effectiveness of the presented technique. In the evaluation, we showed that the PRP performance of the presented technique is superior to that of the other ones despite that the average energy consumption of all the EDs of the presented technique is less than that of the other ones.

## Figures and Tables

**Table 1:** Summary of existing studies.

Reference	Summary
[17]	Distance based allocation for connectivity
[22]	Maximization of minimum throughput of ED
[23]	Minimizing collision probability with transmission power control
[24]	Distance based allocation for maximization of minimum PRP
[25]	Throughput maximization with a fair and unfair allocation
[26]	Maximization of PRP with clustering algorithm
[27]	Distance based allocation with K-means clustering
[28]	Maximization of PRP

**Table 2:** Examples for sensitivity and time on air for a 125 kHz bandwidth, coding rate 4/5, 20 byte payload and 8 symbols at preamble.

Spreading Factor	7	8	9	10	11	12
Sensitivity	−123.0 dBm	−126.0 dBm	−129.0 dBm	−132.0 dBm	−134.5 dBm	−137.0 dBm
Time on Air	61.7 ms	113.2 ms	205.8 ms	370.7 ms	659.5 ms	1318.9 ms

**Table 3:** Current consumption of SX1272 for packet transmission and sleep mode for transmit power 10 dBm.

State	Packet Transmission	Sleep Mode
Current consumption	31 mA	100 nA

**Table 4:** Parameter setup.

Description	Variable	Numerical Value(s)
Placement of EDs	-	uniformly distributed
Number of ED	$N_{\mathrm{ED}}$	Max. 400
Radius of communication area	*R*	6000 m, 10,000 m
Path loss exponent	-	2.7 (suburban)
Transmission power	$P_{\mathrm{TX},i}$	10 dBm
Carrier frequency	-	868.1 MHz
Bandwidth	-	125 kHz
Number of channels used	-	1
Transmission intervals	$T_{i}$	360 s/1 packet
Duty cycle	-	<1%
Simulation period	-	24 h
Number of individuals	$N_{I}$	128
Number of islands	$N_{\mathrm{IS}}$	16
Migration intervals	-	10
Selection	-	elite selection
Crossover	-	two-points crossover
Number of elite	$N_{E}$	2
Probability of mutation	-	0.5
Generation	-	2000

**Table 5:** PRP results according to distance between the LoRa GW and each ED.

Distance	0 m to 1000 m	1000 m to 2000 m	2000 m to 3000 m	3000 m to 4000 m	4000 m to 5000 m	5000 m to 6000 m	Averaged
$W_{C}=1.1I_{\mathrm{MIN}}$	0.999	0.996	0.990	0.981	0.969	0.960	0.983
$W_{C}=1.2I_{\mathrm{MIN}}$	0.999	0.996	0.990	0.983	0.972	0.964	0.984
$W_{C}=1.5I_{\mathrm{MIN}}$	0.999	0.996	0.990	0.984	0.977	0.969	0.986
$W_{C}=1.8I_{\mathrm{MIN}}$	0.999	0.996	0.991	0.985	0.979	0.970	0.987
$W_{C}=I_{\mathrm{MIN}}$	0.999	0.996	0.990	0.980	0.967	0.949	0.980

**Table 6:** Average current consumption results according to distance between the LoRa GW and each ED. Unit for all values is mAh.

Distance	0 m to 1000 m	1000 m to 2000 m	2000 m to 3000 m	3000 m to 4000 m	4000 m to 5000 m	5000 m to 6000 m	Averaged	Constraint
$W_{C}=1.1I_{\mathrm{MIN}}$	19.243	19.263	21.236	20.339	19.413	30.201	21.619	23.372
$W_{C}=1.2I_{\mathrm{MIN}}$	19.275	19.270	24.733	22.821	20.079	33.542	23.297	25.503
$W_{C}=1.5I_{\mathrm{MIN}}$	19.330	20.111	34.531	28.385	24.251	42.732	28.238	31.871
$W_{C}=1.8I_{\mathrm{MIN}}$	19.654	21.740	45.918	34.278	28.294	51.099	33.522	38.262
$W_{C}=I_{\mathrm{MIN}}$	19.285	19.273	19.266	19.262	19.276	25.051	20.238	21.254

**Table 7:** Averaged computation time (sec) of spreading factor allocation under conditions listed in Table 4. $W_{C}=1.5I_{\mathrm{MIN}}$ mAh and R=6000 m. Results are obtained by the computer with specifications: Intel(R) Xeon(R) Gold 6140 CPU@2.30 GHz, memory size is 64 GB.

Number of ED	50	100	150	200	250	300	350	400
Computation time (s)	5.81	11.63	19.11	28.98	40.13	56.18	76.61	96.36
